# Targeting KRAS^G12V^ mutations with HLA class II-restricted TCR for the immunotherapy in solid tumors

**DOI:** 10.3389/fimmu.2023.1161538

**Published:** 2023-05-23

**Authors:** Qi Ai, Fanlu Li, Siyi Zou, Zehui Zhang, Yangbing Jin, Lingxi Jiang, Hao Chen, Xiaxing Deng, Chenghong Peng, Nan Mou, Chenlei Wen, Baiyong Shen, Qian Zhan

**Affiliations:** ^1^ Department of General Surgery, Pancreatic Disease Center, Ruijin Hospital, Shanghai Jiao Tong University School of Medicine, Shanghai, China; ^2^ Research Institute of Pancreatic Diseases, Shanghai Jiao Tong University School of Medicine, Shanghai, China; ^3^ State Key Laboratory of Oncogenes and Related Genes, Institute of Translational Medicine, Shanghai Jiao Tong University, Shanghai, China; ^4^ Department of Cell Therapy, Shanghai Genbase Biotechnology Co., Ltd, Shanghai, China

**Keywords:** KRAS G12V mutation, T cell receptor-engineered-T cell, immunotherapy, human leukocyte antigen-DPB*0301, human leukocyte antigen-DPB*1401, solid tumor

## Abstract

KRAS mutation is a significant driving factor of tumor, and KRAS^G12V^ mutation has the highest incidence in solid tumors such as pancreatic cancer and colorectal cancer. Thus, KRAS^G12V^ neoantigen-specific TCR-engineered T cells could be a promising cancer treatment approach for pancreatic cancer. Previous studies had reported that KRAS^G12V^-reactive TCRs originated from patients’ TILs could recognized KRAS^G12V^ neoantigen presented by specific HLA subtypes and remove tumor persistently *in vitro* and *in vivo*. However, TCR drugs are different from antibody drugs in that they are HLA-restricted. The different ethnic distribution of HLA greatly limits the applicability of TCR drugs in Chinese population. In this study, we have identified a KRAS^G12V^-specific TCR which recognized classII MHC from a colorectal cancer patient. Interestingly, we observed that KRAS^G12V^-specific TCR-engineered CD4^+^ T cells, not CD8^+^ T cells, demonstrated significant efficacy *in vitro* and in xenograft mouse model, exhibiting stable expression and targeting specificity of TCR when co-cultured with APCs presenting KRAS^G12V^ peptides. TCR-engineered CD4^+^ T cells were co-cultured with APCs loaded with neoantigen, and then HLA subtypes were identified by the secretion of IFN-γ. Collectively, our data suggest that TCR-engineered CD4^+^ T cells can be used to target KRAS^G12V^ mutation presented by HLA-DPB1*03:01 and DPB1*14:01, which provide a high population coverage and are more suitable for the clinical transformation for Chinese, and mediate tumor killing effect like CD8^+^ T cells. This TCR hold promise for precision therapy in immunotherapy of solid tumors as an attractive candidate.

## 1 Introduction

Pancreatic ductal adenocarcinoma (PDAC) is mostly diagnosed in advanced stage, and the five-year survival rate is less than 9%, which is a difficult problem in human refractory cancer (1). Although immunotherapy has significant efficacy in many malignant tumors (2–5), for PDAC, many clinical trials such as immune checkpoint inhibitors, cancer vaccines, adoptive cellular immunotherapy and so on show unsatisfactory efficacy, and the key factor is its immunosuppressive tumor microenvironment (6, 7). T cell infiltration is positively correlated with the improvement of PDAC clinical outcome, and the overall survival time of PDAC patients with high effect T cell infiltration is longer (8–10). Preclinical studies have proved the feasibility of isolating and amplifying TIL from PDAC and other solid tumors *in vitro (* 11, 12). Therefore, the therapeutic strategy of using antigen-specific recombinant T cells to accurately target tumor cells to overcome the problem of immunosuppressive microenvironment is a promising treatment. With the development of new antigen identification, single cell sequencing and recombinant TCR construction, adoptive cell therapy (ACT) has become a powerful strategy for the treatment of cancer (13–16). The infusion of a large number of tumor effector T cells specific to neoantigen can accurately clear the tumor. In addition, the injected T cells can also differentiate into memory T cells to maintain effective effector function and achieve the goal of long-term treatment (17). Compared with CAR, TCR can recognize almost all intracellular and cell surface antigens through MHC restriction system, and can detect lower levels of antigens to achieve hypersensitive recognition (18). In addition, under high antigen pressure, despite the restriction of HLA, TCR-T cells have higher expansion efficiency and lower expression of co-suppressor molecules than CAR-T cells (19). Clay TM et al. reported for the first time that transferring TCR gene into PBL of melanoma patients can produce CTL with anti-tumor response *in vitro (* 20). Compared with peripheral blood T cells, natural TIL obtained from resected tumor suspensions or fragments has higher specific T cell concentration, but due to the malignant microenvironment of tumors, TIL is mostly aging, failure and sensitive to apoptosis, which weakens the long-term survival of functional T cells (21, 22). Therefore, the extraction of sensitive and effective effector T cells from TIL and T cell receptor modification may improve the tumor immunosuppressive microenvironment, which can greatly enhance the efficacy of ACT. Many TCR-T products based on CD8^+^ T cells have been tested in clinical trials (23, 24), most of which are aimed at melanoma. Paul F Robbins et al. have demonstrated that adoptive autologous T cells (CD8^+^ T cells account for more than 2/3) transduced by T cell receptor (TCR) targeting NY-ESO-1 can mediate tumor regression in patients with metastatic melanoma and synovial cell sarcoma, with response rates of 45% and 67% respectively (25). In addition to the fact that effector CD8^+^ T cells can be used as the main killing effector cells of recombinant TCR-T cells, CD4^+^T cells have also been shown to excrete cytokines IFN-γ and tumor necrosis factor (TNF) through a variety of mechanisms, thus eliminating tumor cells *in vivo* independently of CD8^+^ T cells to play an anti-tumor effect (26–32). Therefore, it can also construct recombinant TCR as effective memory cells to kill tumor cells. Eric Tran et al. demonstrated that the tumor infiltrating lymphocytes (TIL) of metastatic lung tumors from patients with metastatic cholangiocarcinoma contain CD4^+^ T cells that recognize ERBB2IP mutations expressed in the tumor, and reactive CD4^+^ T cells are dominant. Tumor regression was observed in all lesions after transfusion of ERBB2IP mutation-specific CD4^+^ T cells into the patient. Subsequent experiments showed that these CD4^+^ T cells were effective memory CD4^+^ T cells with cytolytic potential (33).

The selection of appropriate tumor antigens has always been a challenge to construct recombinant engineering T cell targets. KRAS gene is the most frequently mutated oncogene in cancer, 95% of PDAC patients show KRAS oncogene mutations, and KRAS mutations are often associated with poor prognosis and drug resistance of tumors (34, 35). Codon 12 of KRAS, such as G12V, G12D, G12C, etc, has the highest mutation frequency in pancreatic cancer, colorectal cancer and non-small cell lung cancer, accounting for about 90% of all KRAS mutations (35). Among them, KRAS G12V and G12D mutations are the most common, accounting for about 60% of pancreatic cancer, 20% of colorectal cancer, and 8% of non-small cell lung cancer (36, 37). Therefore, KRAS G12V and G12D mutations are ideal targets for PDAC (38–40). Eric Tran et al. found a polyclonal CD8^+^ T cell response to mutant KRAS^G12D^ from tumor infiltrating lymphocytes obtained from a patient with metastatic colorectal cancer, and infused HLA-C*08:02-restricted recombinant TCR-T cells targeting KRAS^G12D^ into the patient, and observed the objective regression of the tumor (41). Rom Leidner et al. treated a patient who had progressive metastatic pancreatic cancer with a single infusion of 16.2×10^9^ autologous HLA-C*08:02–restricted TCR-engineered T cells targeting mutant KRAS G12D, and mediated the objective regression of metastatic pancreatic cancer (42).

Here, we isolated and screened a KRAS^G12V^-specific TCR derived from CD4^+^ T cells of patient’s TILs. Then we identified the HLA restriction of the TCR as HLA-DPB1*03:01 and DPB1*14:01, and tested the neoantigen epitopes and key amino acids of the KRAS^G12V^ mutation. In order to validate that this neoantigen-reactive TCR could recognize KRAS^G12V^ mutation, we constructed the KRAS^G12V^-specific TCR-engineered CD4^+^ T cells and CD8^+^ T cells, and co-cultured them with APC cells and PDAC cell lines loaded with mutant peptides. Notably, we investigated the TCR specificity in functional assays of CD4^+^ T cells but not CD8^+^ T cells, indicating the restriction of this TCR for CD4^+^ T cells. We then validated the antitumor activity of KRAS^G12V^ specific TCR *in vivo* by xenograft mouse model, and found that treatment with KRAS^G12V^-specific TCR could significantly reduce tumor growth. Taken together, we proved the effectiveness of this KRAS^G12V^-specific TCR and expanded the available clinical subtypes of HLA for the development of TCR-based ACT.

## 2 Materials and methods

### 2.1 Cell lines and cell culture

SW620, CFPAC-1, 293T were purchased from ATCC. During the whole duration of this study, all cell lines were regularly tested negative for mycoplasma contamination. SW620 was cultured in RPMI1640 media (Gibco) supplemented with 10% fetal bovine serum (FBS, Gibco), 2 mM L-glutamine, and 1X penicillin/streptomycin (All from Thermo Fisher Science). CFPAC-1 and 293T were cultured in DMEM media (Gibco) supplemented with 10% fetal bovine serum, 2 mM L-glutamine, and 1X penicillin/streptomycin. The cell lines usually kept in culture no more than two months.

### 2.2 Patients and HLA typing of patients

This study was approved by Ethics Committee of Ruijin Hospital of Shanghai Jiao Tong University. Written, informed consent was obtained from all patients. All patients studied were histologically diagnosed with CRC or PDAC and had a confirmed KRAS^G12V^ mutation by whole-exome sequencing (Genergy Bio-Technology, Shanghai). B13 patient was diagnosed with CRC, and the tumor stage of B13 patient is IIIB (T3N1M0). Patient lymphocytes from peripheral blood were genotyped for HLA class II by high-resolution, high-throughput HLA genotyping with deep sequencing (Tissuebank, Shanghai) and found to be HLA-DPB1*03:01 (**Supplementary Table S1**).

### 2.3 Isolation and expansion of TILs

After being washed twice by the Dulbecco’s Phosphate-Buffered Saline (DPBS, Thermo Fisher), fresh tumor samples were chopped into small pieces about 2-4 mm, and then cultured in 24-well plates, with Tumor infiltrating T Lymphocytes (TIL) media [X-Vivo15 (Lonza, BE02-060F) +25 mM Hepes+2% human AB Serum +6000 IU/ml IL2 (All from Thermo Fisher)]. Media would be changed every 2-3 days until Tumor infiltrating T Lymphocytes (TILs) converged to 60%-80% (containing about 0.5-3.0×10^6^ TILs) of one 24-well plate. Amplified TILs were harvested and stored in CryoStor CS10 (Sigma-Aldrich, C2874) cryopreservation solution.

### 2.4 Antigen presentation mRNA or peptide preparation

The whole gene containing mutated KRAS genes (e. g. G12V, G12D) was synthesized and linked to LAMP3 signal peptide (leading sequence) to form LAMP3 LS-KRASmut, added with Xenopus globin UTR at both sides, and then cloned into a pcDNA3.1 plasmid containing prokaryotic T7 promotor, eukaryotic promotor and ORIP replication starting point. mRNA was transcribed by mMessage mMachine™ T7 Transcription Kit (Invitrogen, AM1344) as manufacturer’s instructions *in vitro*, packed in 10 μg/tube, and stored at -80°C. A long peptide of 23-25 length (HPLC, purity > 95%) was synthesized from mutated KRAS, dissolved into 10mg/ml with DMSO, and stored at -80°C.

### 2.5 Antigen-presenting cells and KRAS^G12V^ mRNA loading assay

Peripheral blood mononuclear cells (PBMCs) from patients were isolated by Ficoll-Paque gradient centrifugation and cryopreserved for generating LCLs and DCs. Specifically, 0.5-1.0×10^7^ PBMCs were resuspended in RPMI media supplemented with 10% fetal bovine serum (FBS) and infected with EBV supernatant from the marmoset cell line B95.8 to be induced into immortalized B cells called lymphoblastoid cell line (LCL). During the induction period, half of the culture medium was changed every 7 days. After being cultured for 3 to 4 weeks, the induced LCL were amplified and cryopreserved in cell culture freezing medium. Monocyte-derived immature dendritic cells (DCs) were sorted by CD14 separate magnetic beads using MACS CD14 Isolation Kit (Miltenyi Biotec, 130050201) according to the manufacturer’s instructions, and then cultured in DC induced media [AIM-V medium (Gibco, 12055091) +2% Human AB serum +1000 IU/ml IL4 + 1000 IU/ml granulocyte-macrophage colony-stimulating factor (GM-CSF) (All from Thermo Fisher)]. Induced DCs were cultured in fresh media on the third day and cryopreserved on days 5 to 6. Mature DCs were loaded with mRNA of KRAS G12V mutations by electroporation. In brief, messenger RNA coding KRAS G12V mutation was produced by CHINESE PEPTIDE as instructed by the manufacturer. Mature DCs or LCL were resuspended in Resuspension Buffer R at a finally density of 1.0×10^7^ cells/ml by The Neon™ Kits (MPK10096, Invitrogen), and then added 100 µl cell suspension to the tube containing 5-8 μg mRNA and gently mix. Later, the cell-mRNA mixture was aspirated into the Neon™ Tip and electroporated at 1500 V for one pulse and 30 ms with the Neon™ device (MPK5000, Invitrogen) as instructed by Neon™ Transfection System User Guide. After that, transfected DCs or LCL were resuspended in the complete medium and transferred to the 24-well plate. GFP control was set to assess transfection efficiency by fluorescent microscopy.

### 2.6 Screening of neoantigen-specific T cells and Single-cell TCR sequencing

Transfected DCs or LCLs were used as APC to stimulate TILs for cytotoxic T lymphocyte (CTL) induction. 1.2×10^6^ TILs were co-cultured with pulsed or non-pulsed 0.5×10^5^ DCs cells or 4×10^5^ LCLs in X-Vivo15 medium in 96-well plate. The supernatant of the culture medium was collected after 16 hours of culture, and the release of IFN-γ in supernatant was determined by Human IFN-γ Flex Set (BD Bioscience, 558269) to evaluate T cells’ ability of specifically identifying and killing APCs. Neoantigen-specific T cells were stained with CD3, 41-BB, PI (propidium iodide solution) and analyzed by flow cytometry to sort out PI-/CD3-/41BB+ T cell group. Specifically, 1×10^6^ TILs stimulated by APCs were re-suspended in flow buffer (DPBS solution containing 1% human AB serum and 2 mM EDTA), added with CD3/CD137 antibody and PI, incubated at 4 °C for 1 hour, then washed with flow buffer twice, and sorted by BD FACSAiraII flow sorter. The sorting population was PI-, CD3+ and CD137+. The selected cells were stored in RPMI1640 medium containing 10% human AB serum and placed on ice. The collected sorted cells (PI-, CD3+, CD137+) were centrifuged at 4 °C, 300 g for 10 minutes, then washed with DPBS twice, and re-suspended in DPBS. The sorted T cells were used for the following TCR sequencing analysis by 10X genomics Single Cell Sequencing. For the quality inspection and counting of single cell suspension, the cell survival rate is generally required to be more than 80%. The qualified cells are washed and re-suspended to prepare suitable cell concentration at 700~1200 cells/μl for computer operation of 10X Genomics Chromium™ system. Using 10X Genomics Chromium™ system, gel Bead with sequence tags, sorted T cells and reagent premixed liquid and oil were loaded into their respective injection channels, a “double cross” cross system was formed through the microfluidic channel network to finally emerge a single-cell micro-reaction system GEMs wrapped by oil droplets. The single cell was isolated by gel beads, and then the mRNA molecule was released by cell cleavage and reverse-transcribed into cDNA by the polyT primer. The V (D) J region was amplified by a pair of primers near the 5-terminal UTR region of the V (D) J region and the gene C region. During the amplification process, the unique molecular tag (UMI) of the cell marker and transcript marker were introduced, and the amplified products were digested with restriction endonuclease to produce the second generation sequencing library. Finally, the high-throughput sequencing is carried out by using the double-ended sequencing mode of Illumina platform. The Cell Ranger- mkfastq subroutine was used to convert the sequencing data of BCL format into FastQ format. Using FastQC software to analyze the quality control of preprocessed data. Cell Ranger- VDJ subroutine was used to assemble the V (D) J region sequence of TCR gene of every single cell. IMGT database (https://support.10xgenomics.com/single-cell-vdj) and VDJ database (https://vdjdb.cdr3.net/) were used to analyze the results, and Loupe V (D) J Browser was used for the visualization of analysis results.

### 2.7 Synthesis and verification of recombinant TCR

Single cell sequencing data were analyzed and T cell clones were selected for verification. TCRVα and TCRVβ chains were synthesized and linked to mouse TCR constant region, and P2A (self-cleaving 2A peptide, 2A) sequence was used to link TCRα and TCRβ to form TCRVα-mTCRα-P2A-TCRVβ-mTCRβ structure, which was cloned into lentivirus shuttle vector (GV401) as manufacturer’s instructions. Transfected 293T cells with lentivirus shuttle vector as manufacturer’s instructions to package into lentiviral vector, and harvested supernatants after 24 h and 48 h of incubation. Human peripheral blood lymphocytes from different healthy donors (n=4) were used. PBMC (Sailybio, SLB-HP100B/200365) were activated in 6-well plate coated with CD3 (OKT3) and CD28 (15E8) antibody at 10^6^ cells/ml for 24 hours, then were transduced with lentiviral vector containing TCR and cultured for 6-8 days for TCR screening. The transduced T cells were collected and washed with FACS buffer. 1×10^6^ modified T cells were added with mouse TCRβ constant region antibody for staining to detect the expression of recombinant TCR. Antigen presenting cells and the prepared TCR-T cells were co-cultured as 1:1 in the RPMI1640 medium containing 2% FBS in 96Well-plate overnight. The specific IFN-γ release in the supernatant was determined to verify the specificity of recombinant TCR to Neoantigen.

### 2.8 HLA restriction assay of TCR

Engineered-TCR T cells were incubated with LCLs loaded with neoantigen. The LCLs were from LCL bank (from Shanghai Jikai Genome Medical Technology Co., Ltd) which have been identified HLA restriction for each LCL lines and used as the antigen presenting cell line (**Supplementary Table S3**). Specifically, LCLs from different donors were loaded with KRAS G12V-T15 peptide (TEYKLVVVGAVGV, 10 μg/ml) and co-cultured with Mock T cells and engineered-TCR T cells in RPMI1640 culture medium containing 2% fetal bovine serum at the ratio of 2×10^4^: 2×10^4^, and TCR-T specific IFN-γ release was used to determine HLA restriction.

CIITA gene (Genebank ID: 4261) was overexpressed in SW620 and CFPAC-1 cells by CIITA lentiviral transfection to construct SW620-CIITA and CFPAC-1-CIITA cell lines. After that, HLA-DPB1*03:01 and HLA-DPA1*02:02 was overexpressed in SW620 and CFPAC-1 cells by HLA lentiviral transfection to construct SW620/CFPAC1-CIITA-DPB1*03:01 cell lines and SW620/CFPAC1-CIITA- DPA1*02:02-DPB1*03:01 cell lines. The above cells were collected and suspended in RPMI1640 medium and cultured at 37 °C for 2 hours, then washed twice with DPBS. Antigen presenting cells (modified SW620 or CFPAC-1) and B13.14.1 TCR-T cells were cultured overnight in RPMI1640 medium containing 2% fetal bovine serum as the ratio of 2×10^4^: 2×10^4^, and the release of IFN-γ in the supernatant was determined.

### 2.9 Determination of neoantigen epitopes and key amino acids

Synthetic peptides containing G12V mutant sites (TEYKLVVVGAVG, G12V-T3; YKLVVVGAVGVG, G12V-T6; EYKLVVVGAVG, G12V-T9; YKLVVVGAVG, G12V-T10; KLVVVGAVG, G12V-T11; TEYKLVVVGAV, G12V-T12; EYKLVVVGAV, G12V-T13; YKLVVVGAV, G12V-T14; TEYKLVVVGAVGV, G12V-T15; EYKLVVVGAVGV, G12V-T16; YKLVVVGAVGV, G12V-T17) and alanine substitution (**Supplementary Table S4**) were loaded into autologous LCL cells and co-cultured with TCR-T cells. Neoantigen epitopes and key amino acids were determined by IFN-γ release assay in the supernatant. Specifically, the 9-23 peptides were synthesized and then dissolved in DMSO. The autologous LCL cells of B13 patients were re-suspended in the RPMI1640 culture medium, adding the above peptides to the final concentration of 1 μg/ml. After incubating for 2 hours, they were washed twice with DPBS solution, and then re-suspended with RPMI1640 culture medium containing 2% fetal bovine serum to 2×10^5/^ml. The antigen presenting cells loaded with peptides and B13.14.1 TCR-T cells were cultured overnight according to the ratio of 2×10^4^: 2×10^4^. The release of IFN γ in the supernatant was measured by the Human IFN-γ Flex Set (BD Bioscience, 558269).

By replacing the peptides of KRAS^G12V^ epitopes with alanine one by one, the key amino acids involved in antigen presentation in KRAS^G12V^ epitopes can be screened. Specifically, peptides of KRAS^G12V^ epitopes with alanine replaced were synthesized (**Supplementary Table S4**). IFN-γ or IL2 in the supernatant of antigen presenting cells co-cultured with B13.14.1 TCR-T were determined by the Human IFN-γ Flex Set (BD Bioscience, 558269).

### 2.10 Killing assay of Engineered-TCR T cells

SW620-CIITA-DPB1*03:01 or CFPAC-1-CIITA-DPB1*03:01 cells were re-suspended in RPMI1640 medium containing 2% FBS and inoculated in 96-well plates as 10^4/^well. Mock-T, Engineered-TCR-CD4^+^ T cells and Engineered-TCR-CD8^+^ T cells were added separately according to the proportion of 10:1, 3:1, 1:1, 0.3:1, 0.1:1. After 48 hours of co-incubation, the medium was removed and washed with 200 μl DPBS solution per well. 100 μl RPMI1640 medium containing 2% FBS and 10 μl CCK8 detection reagent (Cell Counting Kit-8, Sigma Aldrich, 96992) were added to each well and incubated at 37 °C for 1 hour to detect 450nm light absorption. Killing rate was:

Specific Lysis%= 1 - (light absorption value/control hole light absorption value)

### 2.11 Engineered-TCR-T cells functional avidity determination

Autologous LCL cells or the SW620-DPB1*03:01 were loaded with different concentrations of KRAS^G12V^-T15 peptide (TEYKLVVVGAVGV) and corresponding wild type peptide (TEYKLVVVGAGGV) (10 μg/ml, 1 μg/ml, 0.1 μg/ml, 0.01 μg/ml, 0.001 μg/ml) at 37 °C for 2 hours, then washed twice with DPBS. The antigen-presenting cells or the SW620-DPB1*03:01 and the engineered-TCR-T cells were co-cultured overnight in RPMI1640 medium containing 2% fetal bovine serum according to the ratio of 2×10^4^: 2×10^4^, and the release of IFN-γ or IL-2 in the supernatant was determined.

### 2.12 IFN-γ and IL-2 determination assay

Antigen presenting cells were loaded with or without mRNA of KRAS G12V mutations, or a range of indicated concentrations of synthetic peptides as previously described. Neoantigen-reactive CD4^+^ T cells clones were incubated with the antigen presenting cells at a ratio of 1:1-1:4 in X-Vivo15 medium overnight. The release of IFN-γ and IL-2 in the supernatant were determined by the Human IFN-γ Flex Set (BD Bioscience, 558269) and Human IL-2 Flex Set (BD Bioscience, 558270). Specifically, the IFN-γ or IL-2 standard (2500 pg/ml) was added with Assay Diluent to dilute by gradient (1: 2, 1: 4, 1: 8, 1: 16, 1: 32, 1: 64, 1: 128, 1: 256), and using 500 μl Assay Diluent as negative control tube (0 pg/ml). The total amount of capture microspheres and antibodies detected by diluted PE markers needed to be diluted in the test were determined according to 50 μl/sample, respectively. The volume of each captured microsphere and PE labeling antibody detection reagent were determined according to 1 μl/sample, respectively. Mix the capture microsphere with the capture microsphere diluent in a tube and mark “mixed capture microsphere”. Dilute the PE antibody detection reagent with the detection antibody diluent, mix it in a tube, and label “mixed PE to detect antibody”. Use the first 4°C to avoid light. 50 μl gradient diluted standard substance was added to each tube of standard quality control, and 50 μl supernatant to be tested in each sample tube. Scrolled mixed microspheres for at least 5 seconds, and added 50 μl mixed microspheres per tube. Mixed gently and incubated at room temperature for 1 hour. Then 50 μl PE labeled antibody was added to each tube and mixed gently and incubated at room temperature for 2 hours. Added 1ml lotion to each tube and centrifuged 200 g for 5 minutes. Carefully absorbed or gently poured out the supernatant, and add 300 μl lotion per tube to resuscitate cells. Samples should be tested as soon as possible after the instrument is adjusted. After the sample was collected (FCS2.0 format), the standard curve was drawn and the data was analyzed by CBA special analysis software FCAPArrayv1.0.

### 2.13 Mouse xenograft models

SW620-CIITA-DPB1-Luc single clone was generated (SW620 was transfected with CIITA, HLA-DPB1*03:01 and firefly luciferase) and then was injected subcutaneously into NOD-scid IL2rγ^null^ (NSG) mice to establish xenograft. TCR-T cells treatment that intravenous injections of 1×10^7^ engineered CD4^+^, CD8^+^ or mixed T cells (CD4^+^: CD8^+^= 1:1) expressing B13.14 TCR separately was given when the tumor size reached about 50 mm^3^ after tumor inoculation. Control mice received no treatment (Mock) T cells. The tumor size was determined by measuring the vertical diameter of each tumor with a caliper, and the calculation formula was as follows: tumor volume (mm^3^) = [(length) × (width) × (width)]/2. On day 9, 16 and 34, the mice were anesthetized by intraperitoneal injection of 0.7% pentobarbital sodium according to the dose of 10 μl/g and were imaged under the living imager to observe the fluorescence.

### 2.14 Statistics

Data analysis was performed using GraphPad Prism v8.0 (GraphPad Software, CA). Data represent mean ± SD of triplicates. Statistical comparison was conducted with Student’s test, and two-way ANOVA.

## 3 Results

### 3.1 Isolation of KRAS^G12V^-reactive T cells from TIL and TCR sequencing of sorted KRAS^G12V^-reactive T cells

**Figure 1:**
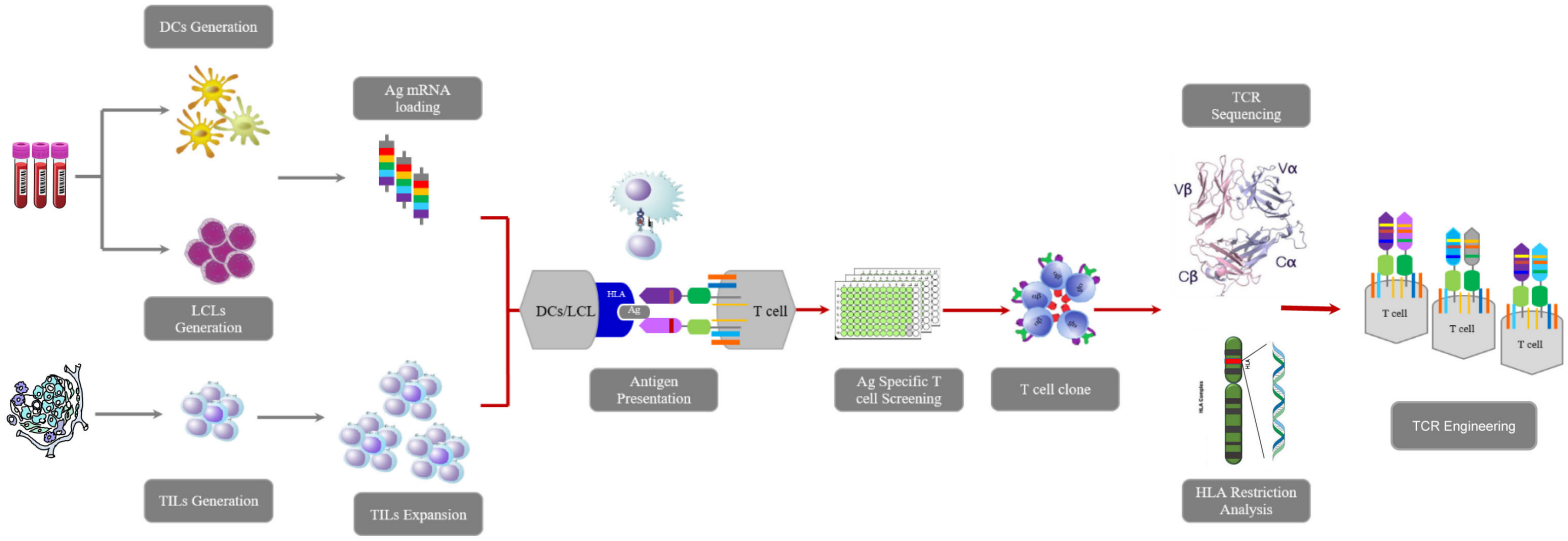
Workflow of this research including TILs isolation, TCR sequencing, tumor-reactive TCR-engineered T cells construction, determination of HLA restriction, tumor killing assay *in vivo and vitro*.

**Figure 2:**
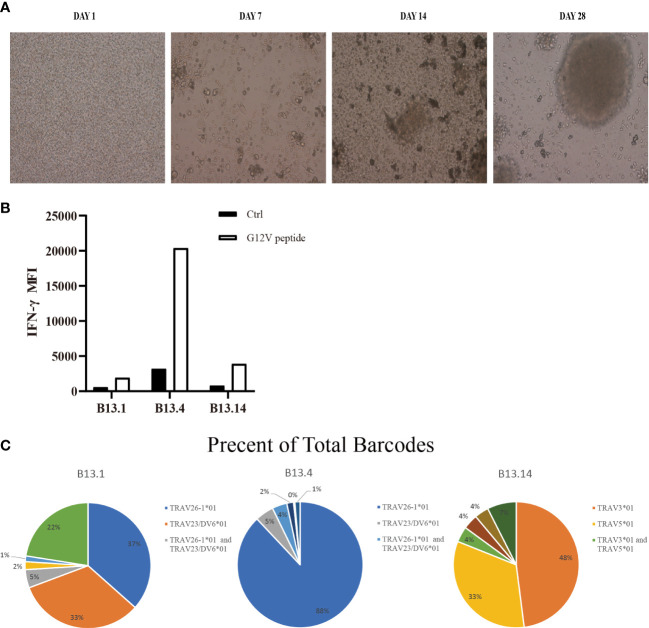
Screen of tumor-reactive TILs and identification of TCRα and TCRβ sequences of sorted CD4^+^ T cells. **(A)** Representative images of the process of LCLs inducement by EBV. **(B)** Flow Cytometry measuring secretion of INF-γ from B13.1, B13.4, B13.14 TILs following the co-culture with LCLs loaded with G12V peptide or wild type peptide. **(C)** Frequencies of top five TILs clonotypes of B13.1, B13.4, B13.14 TILs, respectively.

To obtain KRAS^G12V^-reactive T cells, we collected surgically resected tumor tissues of 16 patients who were diagnosed with CRC and 15 patients who were diagnosed with PDAC as experimental samples for TIL, and obtained their peripheral blood mononuclear cells (PBMCs) for DCs and LCLs (**Figure 1**). PBMCs were induced to lymphoblastoid cell line (LCL) by EBV (**Figure 2A**). TIL clones were subsequently isolated and assessed with their activity and specificity. To screen tumor-reactive TILs, TILs from different tumor specimens were separately co-cultured with mature dendritic cells (DCs) and LCLs loaded with KRAS^G12V^ mRNA. As a result, we found that tumor fractions TIL-B13.1, TIL-B13.4 and TIL-B13.14 from B13 patient who was diagnosed with colorectal cancer produced a significantly higher level of IFN-γ than other TIL fractions and its control group (**Figure 2B**).

B13.1 TILs, B13.4 TILs and B13.14 TILs stimulated by APC cells were sorted by BD FACSAirall flow sorter and then were sequenced their TCRα and TCRβ chains by 10X genomics Single Cell Sequencing. Top three TIL clonotypes were identified, and the percentages of the first ranked TIL clonotypes were 36.55% in B13.1 TILs group, 87.98% in B13.4 TILs group, 47.98% in B13.14 TILs group, respectively (**Figure 2C**). Dominant TCRα and dominant TCRβ chains were identified, TRAV26-1*01-J12*01 was paired with TRBV3-1*01-D1*01-J2-7*01 in B13.1 TILs group, TRAV5*01-J6*01 was paired with TRBV7-9*01-D1*01-J2-1*01 in B13.4 TILs group, and TRAV3*01-J10*01 was paired with TRBV19*01-D1*01-J1-6*02 in B13.14 TILs (**Table 1**), which suggested that the first ranked TCR could be most likely tumor-reactive TCR.

**Table 1:** Top three TCR sequences of B13.1 TILs, B13.4 TILS and B13.14 TILs.

Clonotype	TRAV	TRAJ	TRBV	TRBJ	CDR3α	CDR3β	Clonotype Frequency
B13.1	TRAV26-1*01	TRAJ12*01	TRBV3-1*01	TRBJ2-7*01	CIVRVEGSSYKLIF	CASSQGGSYEQYF	36.55%
	TRAV23/DV6*01	TRAJ58*01	TRBV5-6*01	TRBJ2-7*01	CAASEETSGSRLTF	CASSWNLQASYEQYF	32.75%
	TRAV26-1*01 TRAV23/DV6*01	TRAJ12*01 TRAJ58*01	TRBV3-1*01 TRBV5-6*01	TRBJ2-7*01 TRBJ2-7*01	CIVRVEGSSYKLIF CAASEETSGSRLTF	CASSQGGSYEQYF CASSWNLQASYEQYF	4.68%
B13.4	TRAV5*01	TRAJ6*01	TRBV7-9*01	TRBJ2-1*01	CAEDAGGSYIPTF	CASSLEENEQFF	87.98%
	–	–	TRBV7-9*01	TRBJ2-1*01	–	CASSLEENEQFF	4.79%
	TRAV12-1*01 TRAV5*01	TRAJ7*01 TRAJ6*01	TRBV7-9*01	TRBJ2-1*01	CVVNSLWEQQTRF CAEDAGGSYIPTF	CASSLEENEQFF	3.82%
B13.14	TRAV3*01	TRAJ10*01	TRBV19*01	TRBJ1-6*02	CAVRDGRGGGNKLTF	CASSPGQRDNSPLHF	47.98%
	TRAV5*01	TRAJ6*01	TRBV7-9*01	TRBJ2-1*01	CAEDAGGSYIPTF	CASSLEENEQFF	33.06%
	TRAV3*01 TRAV5*01	TRAJ10*01 TRAJ6*01	TRBV7-9*01	TRBJ2-1*01	CAVRDGRGGGNKLTF CAEDAGGSYIPTF	CASSLEENEQFF	4.00%

### 3.2 KRAS^G12V^-reactive TCR-engineered T cells recognized and killed HLA-DPB1*03:01 tumor cells loaded with KRAS^G12V^

**Figure 3:**
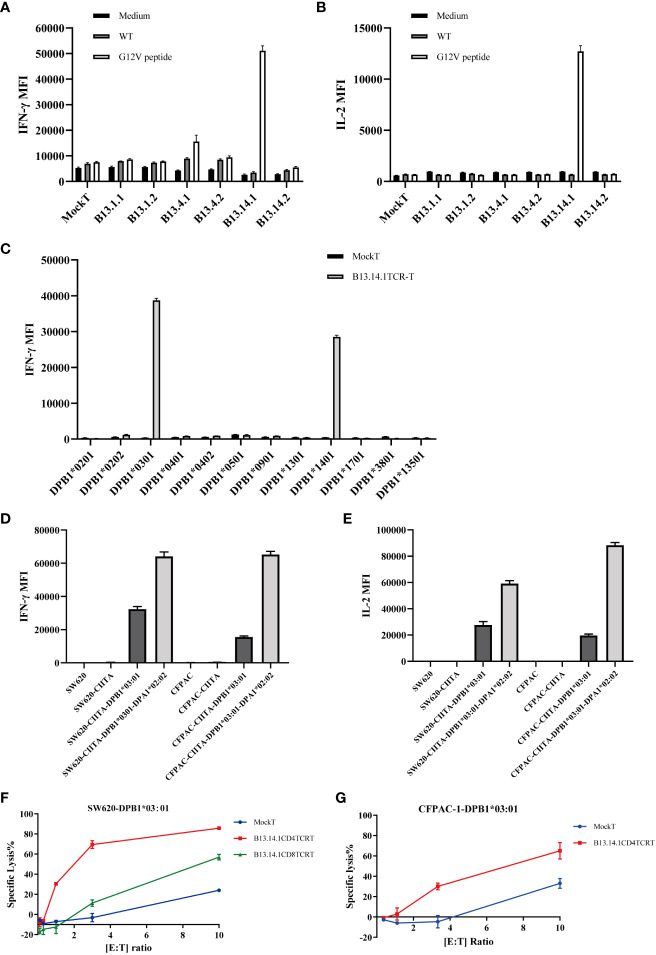
Function evaluation and HLA restriction determination of B13.14.1 TCR-CD4^+^ T cells. **(A, B)** Flow Cytometry measuring secretion of INF-γ and IL-2 from B13.14.1 TCR-engineered T cells or Mock T cells stimulated by autologous LCLs electroporated with KRAS^G12V^ mRNA. **(C)** Flow Cytometry measuring secretion of INF-γ from B13.14.1 TCR-CD4^+^ T cells or Mock T cells stimulated by EBV-LCLs from LCL bank loaded with KRAS^G12V^ mRNA. **(D, E)** Flow Cytometry measuring secretion of INF-γ and IL-2 from B13.14.1 TCR-CD4^+^ T cells or Mock T cells stimulated by SW620, SW620-CIITA, SW620-CIITA-DPB1*03:01, SW620-CIITA-DPA1*02:02-DPB1*03:01, CFPAC-1, CFPAC1-CIITA, CFPAC1-CIITA-DPB1*03:01 and CFPAC1-CIITA-DPA1*02:02-DPB1*03:01, respectively. For **(A–E)**, each graph shows the mean results of three technical replicates from one donor, and each experiment has been performed with a different donor. **(F, G)** Specific lysis of SW620-DPB1*03:01 and CFPAC-1-DPB1*03:01 induced by B13.14.1 TCR-CD4^+^ T cells under different effector cell/target cell ratios. Five different ratios (10:1, 3:1, 1:1, 1:3, and 1:10) were examined.

To obtain KRAS^G12V^-reactive TCR-T cells, the first ranked TCRs were used for function validation for each TIL group (**Table 1**). KRAS^G12V^-reactive TCR gene were synthesized in the order of TRAVmCa-P2A-TRBVmCb as the results of 10X genomics Single Cell Sequencing (**Table 2**) to construct recombinant TCR modified T cells. The modified TCR-T cells were added with mouse TCRβ constant region antibody for staining to detect the expression of recombinant TCR, and the results showed that all 6 recombined TCRs from patient B13 could indeed be expressed in allogeneic T cells (**Supplementary Figure S1**). To evaluate the ability of these TCR-T cells to specifically identify and mediate killing functions in response to KRAS^G12V^ *in vitro*, We performed a co-culture of recombinant TCR modified T cells with autologous LCL cells which were electroporated with KRAS^G12V^ mRNA, and the results showed that B13.14.1 TCRs could specifically recognize KRAS G12V mutation but not wild type KRAS (**Figures 3A, B**).

**Table 2:** TCR sequences of recombinant TCR modified T cells.

Clone Name	TCRα	TCRβ
**B13.1.1**	TRAV26-1*01, TRAJ12*01	TRBV3-1*01, TRBJ2-7*01
**B13.1.2**	TRAV23/DV6*01, TRAJ58*01	TRBV5-6*01, TRBJ2-7*01
**B13.4.1**	TRAV5*01, TRAJ6*01	TRBV7-9*01, TRBJ2-1*01
**B13.4.2**	TRAV12-1*01, TRAJ7*01	TRBV7-9*01, TRBJ2-1*01
**B13.14.1**	TRAV3*01, TRAJ10*01	TRBV19*01, TRBJ1-6*02
**B13.14.2**	TRAV5*01, TRAJ6*01	TRBV7-9*01, TRBJ2-1*01

The patient was genotyped and found to be HLA-DPB1*03:01 (**Supplementary Table S1**). SW620 (colorectal cancer lymph node metastasis) contains KRAS^G12V^ homozygous mutation, and CFPAC-1 cell line (pancreatic cancer) contains KRAS^G12V^ heterozygous mutation. Their HLA-DP matching is shown in the table (**Supplementary Table S2**). In order to verify the HLA restriction, we performed a co-culture experiment using EBV-LCL cells (**Supplementary Table S3**) from LCL bank with B13.14.1 TCR-engineered T cells or mock T cells, the results suggested that the B13.14.1 TCR can recognize the KRAS^G12V^ antigenic peptides presented not only by HLA-DPB1*03:01 but also by HLA-DPB1*14:01 (**Figure 3C**). To verify that the KRAS^G12V^ peptides are endogenously processed and presented on the cells with corresponding HLA molecules, we overexpressed CIITA gene and HLA gene in SW620 and CFPAC1 (**Supplementary Table S2**, **Supplementary Figure S2**), and then co-cultured B13.14.1 TCR-engineered T cells or mock T cells with SW620, SW620-CIITA, SW620-CIITA-DPB1*03:01, SW620-CIITA-DPA1*02:02-DPB1*03:01, CFPAC-1, CFPAC1-CIITA, CFPAC1-CIITA-DPB1*03:01 and CFPAC1-CIITA-DPA1*02:02-DPB1*03:01, respectively. We here demonstrated that B13.14.1 TCR can recognize the restricted G12V mutation presented by HLA-DPB1*03:01, and can be presented better with both HLA-DPB1*03:01 and HLA-DPA1*02:02 (**Figures 3D, E**).

To further evaluate the killing efficiency and characteristics of B13.14.1 TCR-engineered T cells, SW620/CFPAC1-CIITA-DPA1*02:02-DPB1*03:01 were co-cultured with Mock T cells, B13.14.1 TCR-engineered CD4^+^ T cells, and B13.14.1 TCR-engineered CD8^+^ T cells, respectively, with different effector/target cell ratios, i.e., 10:1, 3:1, 1:1, 1:3, and 1:10. The results showed that B13.14.1 TCR-engineered T cells could kill tumor cells in a dose-dependent manner and the killing effect of CD4^+^ T cells was superior to CD8^+^ T cells, indicating its dependence on CD4 (**Figures 3F, G**).

### 3.3 Identification of KRAS^G12V^ mutation epitopes and key amino acids

**Figure 4:**
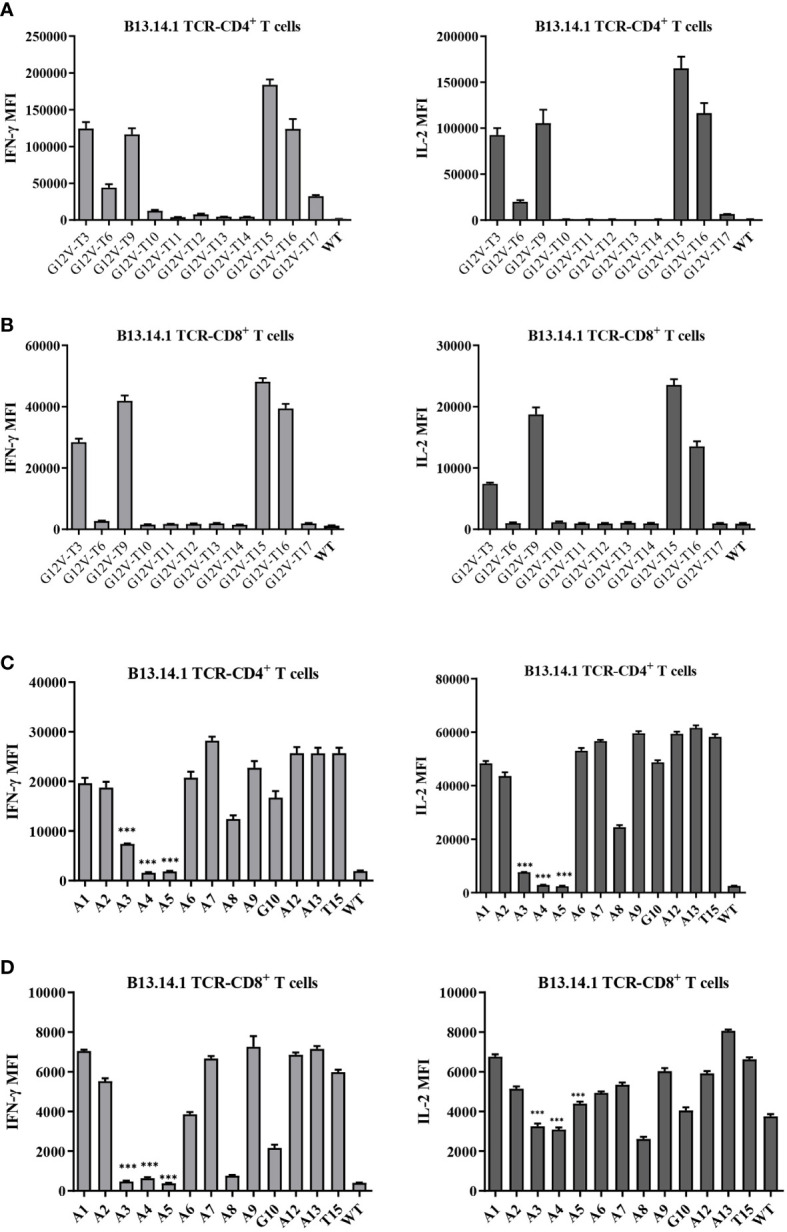
Amino acid residues E (position 2) and G (position12) of the KRAS^G12V^ mutant peptides were essential for G12V epitope presentation and immunoreactivity. **(A, B)** Flow Cytometry measuring secretion of INF-γ and IL-2 from B13.14.1 TCR-CD4^+^/CD8^+^ T cells stimulated by autologous LCLs loaded with 12 peptides. **(C, D)** Flow Cytometry measuring secretion of INF-γ and IL-2 from B13.14.1 TCR-CD4^+^/CD8^+^ T cells stimulated by autologous LCLs loaded with G12V-T15 peptides which were sequentially replaced with alanine (i.e., “A”) from position 1 to 13. Data represent mean ± SD of triplicates. ***p ≤ 0.001.

To further characterize the KRAS^G12V^ mutation epitopes, 9-23 peptides containing G12V mutation were synthesized (**Table 3**) and loaded in the autologous LCLs to present the antigen to the B13.14.1 TCR-engineered T cells. The results of the release of the IFN-γ and IL-2 showed that G12V-T3, G12V-T9, G12V-T15 and G12V-T16 peptides could effectively induce IFN-γ release from B13.14.1 TCR-engineered T cells, of which G12V-T15 is the most effective, indicating that HLA-DPB1*03:01 could present the above peptides (**Figures 4A, B**). The results also suggested that the peptide containing amino acid residues E (position 2) and G (position12) of the KRAS^G12V^ mutant peptides were essential for G12V epitope presentation and could effectively activate T lymphocytes and induce immune response to tumors with KRAS^G12V^ mutations.

**Table 3:** List of KRAS G12V mutation peptides.

No.	Length	Amino acid sequences
**G12V-T1**	23	TEYKLVVVGAVGVGKSALTIQLI
**G12V-T2**	15	AVGVGKSALTIQLI
**G12V-T3**	12	TEYKLVVVGAVG
**G12V-T4**	11	VVVGAVGVGKS
**G12V-T5**	15	VGAVGVGKSALTIQ
**G12V-T6**	12	YKLVVVGAVGVG
**G12V-T7**	15	VVVGAVGVGKSALT
**G12V-T8**	12	LVVVGAVGVGKS
**G12V-T9**	11	EYKLVVVGAVG
**G12V-T10**	10	YKLVVVGAVG
**G12V-T11**	9	KLVVVGAVG
**G12V-T12**	11	TEYKLVVVGAV
**G12V-T13**	10	EYKLVVVGAV
**G12V-T14**	9	YKLVVVGAV
**G12V-T15**	13	TEYKLVVVGAVGV
**G12V-T16**	12	EYKLVVVGAVGV
**G12V-T17**	11	YKLVVVGAVGV
**G12V-T18**	14	TEYKLVVVGAVGVG
**G12V-T19**	16	TEYKLVVVGAVGVGK
**G12V-T20**	16	TEYKLVVVGAVGVGKS
**G12V-T21**	17	TEYKLVVVGAVGVGKSA

Next, the key amino acids involved in antigen presentation in KRAS^G12V^ epitopes was screened by replacing the peptides of KRAS^G12V^ epitopes sequentially with alanine (i.e., “A”) from position 1 to 13 (**Supplementary Table S4**). Investigation of the reactivity of B13.14 TCR-T cells against the autologous LCL cells loaded with these peptides revealed that the ability of B13.14.1 TCR-T cells to recognize KRAS G12V epitopes decreased after p3Y, p4K, p5L and p8V were mutated to alanine, especially p3Y, p4K and p5L (**Figures 4C, D**). Thus, these amino acid (p3Y, p4K, p5L and p8V) may be critical for DPB1*03:01 binding or TCR recognition to DPB1*03:01 and KRAS^G12V^ peptide complex, but the binding of the peptides to the MCH II remains to be verified.

### 3.4 High functional avidity of the KRAS^G12V^-reactive TCR-engineered T cells to KRAS^G12V^ mutants

**Figure 5:**
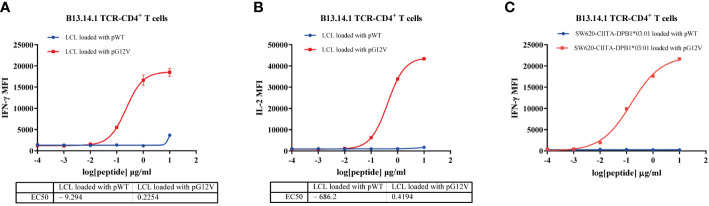
Functional avidity analysis of B13.14.1 TCR-CD4^+^T cells. **(A, B)** Flow Cytometry measuring secretion of INF-γ and IL-2 from B13.14.1 TCR-CD4^+^T cells stimulated by autologous LCLs loaded with different concentrations of KRAS G12V-T15 peptide (TEYKLVVVGAVGV) and corresponding wild type peptide (TEYKLVVVGAGGV), i.e., 10 μg/ml, 1 μg/ml, 0.1 μg/ml, 0.01 μg/ml, 0.001 μg/ml. **(C)** Flow Cytometry measuring secretion of INF-γ from B13.14.1 TCR-CD4^+^ T cells stimulated by SW620-CIITA-DPB1*03:01 with different concentrations of KRAS G12V-T15 peptide (TEYKLVVVGAVGV) and corresponding wild type peptide (TEYKLVVVGAGGV), i.e., 10 μg/ml, 1 μg/ml, 0.1 μg/ml, 0.01 μg/ml, 0.001 μg/ml. Data represent mean ± SD of triplicates.

To further investigate the functional characteristics of B13.14.1 TCR-engineered T cells, we carried out a tumor cell line killing assay with autologous LCL cells or SW620-CIITA-DPB1*03:01 loaded with different concentrations of KRAS G12V-T15 peptide (TEYKLVVVGAVGV) and corresponding wild type peptide (TEYKLVVVGAGGV), i.e., 10 μg/ml, 1 μg/ml, 0.1 μg/ml, 0.01 μg/ml, 0.001 μg/ml. The killing efficacy is positively correlated with the concentrations of the peptide, and the B13.14.1 TCR-engineered T cells can recognize KRAS^G12V^ peptide loaded on autologous LCL cells and SW620-CIITA-DPB1*03:01 at a low concentration of <10 ng/ml (**Figures 5A–C**). Therefore, B13.14.1 TCR-engineered T cell could specifically secrete IFN-γ and IL-2 on encountering the antigen presenting cells in a dose-dependent manner, and it had high functional avidity and high specificity for KRAS^G12V^ mutants.

### 3.5 Treatment efficacy of KRAS^G12V^-reactive TCR-engineered T cells in xenograft model

**Figure 6:**
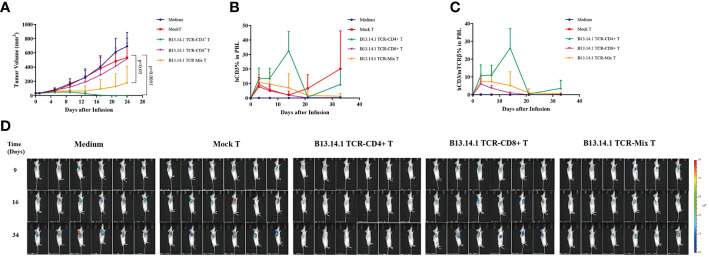
Evaluation of the activities of B13.14.1 TCR-engineered T cells *in vivo*. SW620-CIITA-DPB1*03:01 were implanted into immunodeficient NSG mice and treated them with intravenous injection of PBS, human CD4^+^ T cells, CD8^+^ T cells and the mixture of CD4^+^ T cells and CD8^+^ T cells (CD4^+^ T cells: CD8^+^ T cells = 1:1) transduced with B13.14.1 TCR (TRAV3*01-J10*01/TRBV19*01-D1*01-J1-6*02) when tumor size reached about 50 mm^3^. The results showed **(A)** tumor sizes, **(B)** proportions of human CD3^+^ T cells in the peripheral blood, **(C)** proportions of human CD3^+^ T cells/mouse TCRβ in the peripheral blood. **(D)** Micrographs obtained from IVIS of mice inoculated with SW620-CIITA-DPB1*03:01 and were treated or untreated with Mock T cells or B13.14.1 TCR-engineered T cells.

To test whether B13.14.1 TCR-engineered T cells had antitumor functions *in vivo*, we implanted subcutaneously the KRAS^G12V^-positive SW620-CIITA-DPB1-luc into NOD-scid IL2rγ^null^ (NSG) mice. When tumor size reached about 50 mm^3^, we treated them with B13.14.1 TCR transduced human CD4 T cells, CD8 T cells and the mixture of CD4 T cells and CD8 T cells intravenously, and set intravenous injection of Mock-T (untransduced T cell) and PBS as control groups. Recombinant TCR expression was determined by mTCRβ expression (**Supplementary Figure S3**). The results showed that the growth of tumors in mice treated with B13.14.1 TCR-engineered CD4^+^ T cells was significantly suppressed compared with all control groups (compared with treatment with Mock T cells; P < 0.0001; **Figures 6A, D**). Through tacking B13.14.1 TCR-engineered T cells using human CD3 and mouse TCR constant region TCRβ antibody, we found that B13.14.1 TCR transduced CD4 T cell showed significantly higher expansion *in vivo* than other groups (compared with treatment with B13.14.1 TCR-engineered mix T cells; P = 0.0024; **Figures 6B, C**). The results indicated that B13.14.1 TCR-engineered T cells could inhibit the growth of SW620-DPB1*03:01 tumor, and exhibited the dependence on CD4.

## 4 Discussion

Adoptive TILs and CAR-T transfer-based immunotherapy is a promising therapeutic approach to eliminate a variety of tumors (43–45). However, the validity of adoptive cell therapy for most patients with solid tumors has not yet been fully proved (46, 47). PDAC is a highly immunosuppressive cancer type, and its TILs in the tumor immune microenvironment is often depleted and senile. Hence, the generation *in vitro* of TCR-engineered T cells targeting tumor-specific neoantigens would be a promising strategy to overcome the immunosuppressive status. Recent clinical researches on TCR-T therapy have promising results for curative tumor regression in solid tumors (25, 41, 42, 48). Currently, the choice of tumor-specific neoantigens for antitumor TILs is still a challenge. RAS mutations are the most frequent proto-oncogene mutations, in which KRAS mutation has the highest incidence in solid tumors (about 86% of the three RAS mutations), i.e., approximately 90% in pancreatic cancer and 40% in colorectal cancer (49, 50). About 60%-80% of KRAS mutations are found to be G12V and G12D in pancreatic cancer, and 20%-30% in colorectal cancer (51, 52). Therefore, KRAS^G12V^ may be a good target for pancreatic cancer. It has been reported that the frequency of the HLA-DPB1*03:01 in the Chinese Han population is approximately 3.5913% (N=4845), which ranks the 7^th^ in frequency of DPB1 (53). HLA-DPB1*03:01 and DPB1*14:01 is 3rd rank of HLA-DPB1 alleles in Asian and Caucasian with 20-30% frequency. Also, HLA-DPB1*14:01 is the highest DPB1 alleles found in south Americans (>50%). In this study, we isolated and identified the HLA-DPB1*03:01-restricted human TILs from patient’s tumor tissue that recognize natural processed and presented epitopes in KRAS^G12V^ mutants, and cloned the TCRs to construct KRAS^G12V^-reactive TCR-engineered CD4^+^ T cells. The reason why the specific reaction TCR of KRAS^G12V^ mutation was not isolated in other tumor tissue samples may be that the infiltration density of TIL in tumor tissue samples is too low or the TIL itself is in a suppressed state with low activity, which is not enough to exert anti-tumor killing effect.

Our study not only obtained KRAS^G12V^-reactive TILs but identified and isolated corresponding KRAS^G12V^-reactive TCRs by single-cell TCR sequencing. In addition, B13.14.1 TCR could express stably in primary T cells and mediate the specific recognition of KRAS^G12V^-HLA-DPB1*03:01 complex, leading to stronger antitumor response of efficient killing of target cells. However, the recombined TCR-T cells from B13.1 TILs and B13.4 TILs showed low secretion of IFN-γ, the reason may be that the sequences of reactive TCRs were not be founded by the 10X genomics Single Cell Sequencing. We also demonstrated that B13.14.1 TCR could recognized an additional HLA-DPB1 allele, HLA-DPB1*14:01, which can therefore broaden application of our TCR. The sequences of HLA-DPB1*14:01 and DPB1*03:01 differ by only one amino acid, which may explain why the TCR can respond to these two allotypes. The results of the high functional avidity of our TCR further supported its clinical efficacy. Besides, we compared the functional avidity of B13.14.1 TCR with 6F9 TCR which recognizes MAGEA3 p243-258 in HLA-DPB1*04:01 restricted manner (54) and founded that functional avidity of B13.14.1TCR is comparable with 6F9 TCR in the same assay format (peptide titrated on autologous LCL line). Interestingly, we found that the B13.14.1 TCR-engineered CD4^+^ T cells had higher killing effect than CD8^+^ T cells *in vivo and vitro*. In the immunodeficient mouse models, we found that the response of engineered-TCR CD4^+^ T cells were more effective in antitumor immunity than CD8^+^ T cells or mix T cells. The reason may be that the B13.14.1 TCR is derived from CD4^+^ T cells.

Unlike MHC I which is broadly expressed in majority of tissues and tumor types, expression of MHC II is limited to antigen presenting cell, such as DC and B cell reported in previous publications. Some researches have showed the evidence of MHC II expression in normal tissue and different tumor types. Evidence of MHC II expression in normal tissue was concluded from that HLA-A, B, C, DRB1 and DQB1 matched unrelated hematopoietic cell transplantation patients with HLA-DPB1 mismatch informs risk of GVHD development (55). HLA-DPB1-mismatched donor-derived TCRs can recognize human autoantigens presented in recipient tissue HLA-DPB1, implying that HLA-DPB1 is expressed to a certain extent in recipient normal tissues. MHCII expression was reported in various tumor types originated from different tissues, such as melanoma, breast cancer, colorectal cancer, ovarian cancer, prostate cancer and NSCLC (56). In recent studies, expression of MHC II expression was reported in 77.7% of PDAC (49/63) using immunohistochemistry and half of tumor cells are expressing MHC II at moderate or high levels (57).

Previous studies on TCR-T cell therapy mostly developed MHC class I-restricted TCRs (58) and applied CD8^+^ T cells for killing cells. However, immunotherapies based on CD8^+^ T cells have shown the transient and weak immune response in most patients (59). Some studies showed evident clinical adverse events of CD8^+^ T cell-therapies despite of its remarkable clinical responses (60, 61). In the meanwhile, the last 5 years have seen many reports recognizing the critical role of CD4^+^ T cells driving anti-tumor immunity and in supporting anti-tumor CD8^+^ T cell responses (62). CD4^+^ T cell can infiltrate and recognize autologous tumor in a MHC II-restricted manner (28). High infiltration of CD4^+^ T cell was associated with improved survival in pancreatic cancer patients while CD8^+^ T cell infiltration didn’t have an impact on overall survival (63). Furthermore, cytotoxic CD4^+^ T cells in tumors predicts a clinical response in 244 metastatic bladder cancer patients with anti PDL1 therapy (64). Besides, single cell analysis reveals a CD4^+^ T cell cluster is correlated with efficacy of PD1 blockade in NSCLC (65).

Neoantigen specific T cell may be the main player against human cancer, for example PD1/PDL1 blockade can restore antitumor activity of neoantigen specific tumor infiltrating lymphocytes in solid tumor. Evidence of antitumor activity of neoantigen specific CD4^+^ T cell has been reported in different tumor types including breast cancer (33), melanoma (66), pancreatic cancer (42). Moreover, adoptive CD4^+^ T cell therapy using HLA-DPB1*0401 restricted TCR recognizing MAGEA3 has been proved to be effective in 17 patients with metastatic cancer and 4/17 patients treated with DPB1*0401 restricted TCR-T cells showed durable clinical response (1CR, 3PR) (67). Proteasome processing of tumor antigens and presentation in MHC II are critical for tumor recognition by CD4^+^ T cells. Lysosomal processing and presenting on MHC II of acquired exogenous antigen by tumor cells were considered to be the major antigen presentation pathway of human CD4^+^ T cells (68). However, some studies showed that MHC II can efficiently present intracellular protein to human CD4^+^ T cell in non-classical antigen processing pathways (69, 70). Interestingly, 80% of neoantigen specific TCRs can recognize autologous tumor cells in MHC II restricted manner which were isolated from 62 of 75 gastrointestinal cancer patients (71). A high proportion of MHC II-restricted neoantigen-specific TCRs can be isolated in more than 80% of different tumor types, suggesting that MHC II-presented neoantigens may be more prevalent in human cancers than previously realized. These studies have shown that CD4^+^ T cells can promote the killing of tumor cells in various ways.

In addition to the KRAS^G12V^ mutation, we also conducted experiments on the reactivity of B13.14.1 TCR to other KRAS mutants (G12A, G12D, G12I, G12V, G12S). The result turned out that B13.14.1 TCR showed specific reactivity to KRAS^G12V^ and KRAS^G12I^ but not to other KRAS mutants. It seems that longer methyl group of Isoleucine may enhance interaction of TCR and pMHC complex compared with Valine residue. KRAS^G12D^ accounts for the top one KRAS mutation in solid tumor. We also tried to screen KRAS^G12D^ reactive TCR clone from TIL of patients. However, none of screened T cell clone showed specific KRAS^G12D^ recognition. Dr Steven Rosenberg’s group reported isolation of KRAS^G12D^ specific TCR from CRC patients which was HLA-C*0802 restricted (41), but HLA-C*0802 restricted TCR is not that of valuable for further development because of low frequency of HLA-C*0802 in most countries.

We reported here that B13.14.1 TCR-engineered CD4^+^ T cells were able to recognize and kill MHC class II expressing target cells such as SW620-CIITA-DPB1*03:01 and CFPAC-1-CIITA-DPB1*03:01, in a dose-dependent manner, independent of APCs, which was the same as described in other studies (28, 32). Besides, CD4^+^ T cells can secret multiple cytokines to mediate anti-tumor effects, for instance, the secretion of IL-2 from Th1 cells is essential for the function of CD8^+^ T cells such as the initiation of the immune response and its growth (72). Thus, it is significant to strength the study of the mechanism of CD4^+^ T cells recognizing and killing tumor cell to obtain optimal antitumor responses. The identification of CD4^+^ TCR specific for KRAS mutations provides the foundation for applying the CD4^+^ T-cell immunity to the adoptive transfer therapy and deepens the mechanism of CD4^+^ T cells in human anti-tumor immunity. In the future studies, we would enroll patients with advanced PDAC for clinical researches to validate our approach. However, it is still a challenge to improve the anti-tumor efficacy of TCR-T immunotherapy, including how to expand the range of available TCRs in more patients, how to increase the safety and the functional avidity of therapeutic TCRs, and how to overcome the immunosuppressive effects of suppressor cell subsets in CD4^+^ T cells. With the development of next-generation sequencing technology, TCR-T immunotherapy for personalized neoantigens will become more and more mature in the future and become a viable and ideal cancer treatment.

## Data availability statement

The original contributions presented in the study are publicly available. This data can be found here: GSE232897 (GEO).

## Ethics statement

The studies involving human participants were reviewed and approved by Ethics Committee of Ruijin Hospital of Shanghai Jiao Tong University. The patients/participants provided their written informed consent to participate in this study. The animal study was reviewed and approved by Ethics Committee of Ruijin Hospital of Shanghai Jiao Tong University.

## Author contributions

QZ, BS and CW planned the research and contributed to conception and design of the study, and provided funding support. QA and FL contributed to conception and design of the study, execution of the experiments, data collection, data analysis, and preparation of the manuscript. QA and FL contributed equally to this work and should be considered as co-first authors. SZ, ZZ, YJ, HC, CP and XD have involved in the interpretation of data. LJ and NM guided the revision of this article. NM provided experimental platform and technical support. QZ, CW and BS contributed to the review of the manuscript. All authors contributed to the article and approved the submitted version.
