# Supplementary material for: Targeting KRASG12V mutations with HLA class II-restricted TCR for the immunotherapy in solid tumors
Source: Front Immunol. 2023 May 23;14:1161538. doi: 10.3389/fimmu.2023.1161538 (PMC10243368; doi:10.3389/fimmu.2023.1161538)
Supplement: Supplementary file 4 [file DataSheet_4.docx]

Supplementary Material

Targeting KRAS^G12V^ mutations with HLA class II-restricted TCR for the Immunotherapy in solid tumors

**Qi Ai****^1,2,3#^, Fanlu Li^1,2,3#^, Siyi Zou^1,2,3^, Zehui Zhang^1,2,3^, Yangbing Jin^1,2,3^, Lingxi Jiang^1,2,3^, Hao Chen^1,2,3^, Xiaxing Deng^1,2,3^, Chenghong Peng^1,2,3^, Nan Mou^4*^, Chenlei Wen^1,2,3*^, Baiyong Shen^1,2,3*^ and Qian Zhan^1,2,3*^**

^1^Department of General Surgery, Pancreatic Disease Center, Ruijin Hospital, Shanghai Jiao Tong University School of Medicine, Shanghai, China.

^2^Research Institute of Pancreatic Diseases, Shanghai Jiao Tong University School of Medicine, Shanghai, China.

^3^State Key Laboratory of Oncogenes and Related Genes, Institute of Translational Medicine, Shanghai Jiao Tong University, Shanghai, China.

^4^Department of Cell Therapy, Shanghai Genbase Biotechnology Co.,Ltd, Shanghai, China.

# These authors contributed to the work equally and should be regarded as co-first authors.

*** Correspondence:**Nan Mou

[nan.mou@genbase.com.cn](mailto:nan.mou@genbase.com.cn)

Chenlei Wen

[tianzewcl@hotmail.com](mailto:tianzewcl@hotmail.com)

Baiyong Shen

[shenby@shsmu.edu.cn](mailto:shenby@shsmu.edu.cn)

Qian Zhan

[zhanxi80@126.com](mailto:zhanxi80@126.com)

# Supplementary Data

Supplementary Material should be uploaded separately on submission. Please include any supplementary data, figures and/or tables.

Supplementary material is not typeset so please ensure that all information is clearly presented, the appropriate caption is included in the file and not in the manuscript, and that the style conforms to the rest of the article.

# Supplementary Figures and Tables

For more information on Supplementary Material and for details on the different file types accepted, please see [here](https://www.frontiersin.org/guidelines/author-guidelines#supplementary-material).

## Supplementary Figures


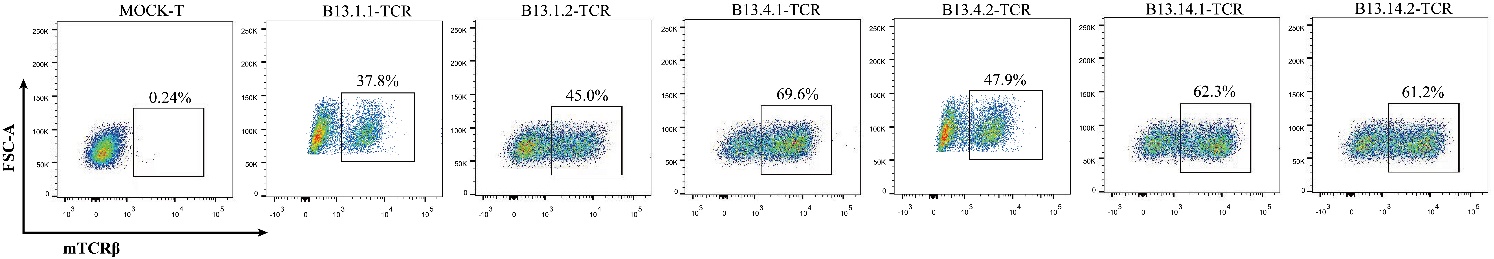


**Supplementary Figure 1.** Expression of recombined TCR after transduction to the T cells by staining with anti-mTCRβ antibodies. Data shown are representative flow diagrams from two or three independent experiments.


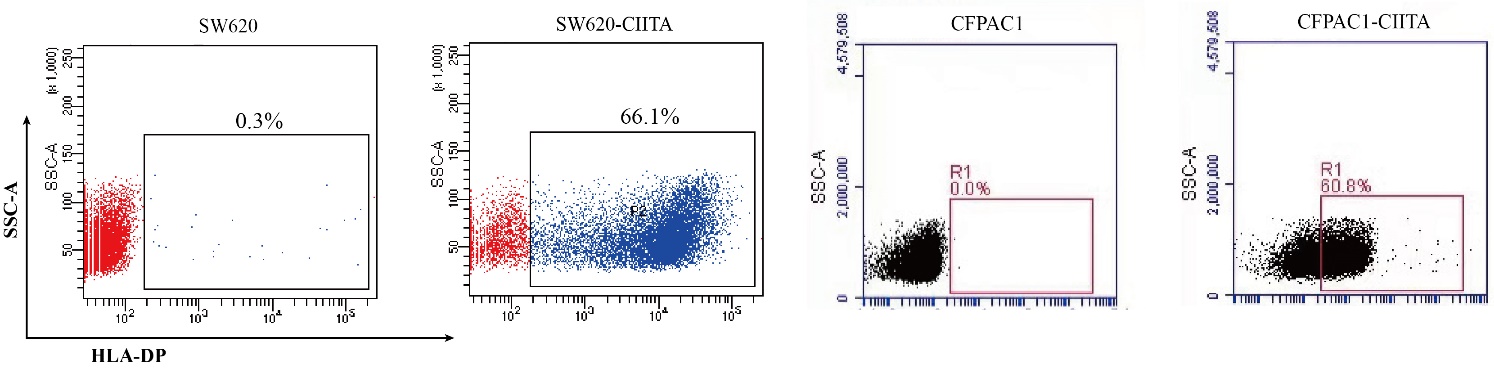


**Supplementary Figure 2.** HLA-DP detection in SW620-CIITA and CFPAC1-CIITA by staining with anti-HLA-DP antibodies. Data shown are representative flow diagrams from two or three independent experiments.


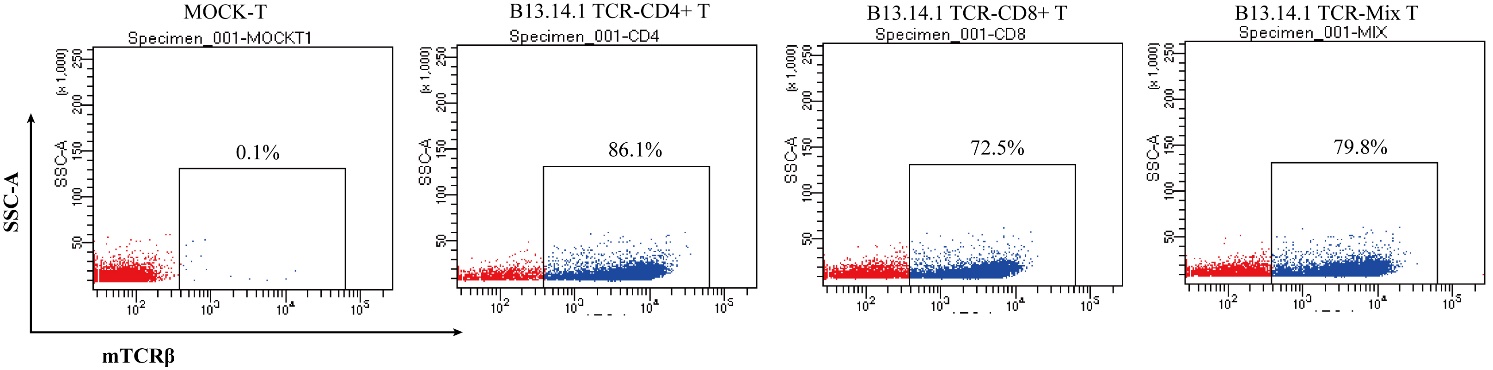


**Supplementary Figure 3.** TCR detection in recombined TCR-T cells by staining with anti-mTCRβ, anti-CD4 and anti-CD8 antibodies. Data shown are representative flow diagrams from two or three independent experiments.

## Supplementary Tables

**Table S1. HLA types of the B13 patient**

| Patient | Classical HLA | | | | | | | | |
| --- | --- | --- | --- | --- | --- | --- | --- | --- | --- |
|  | A | B | C | DRA | DRB1 | DPA1 | DPB1 | DQA1 | DQB1 |
| B13 | 24:02 | 44:03 | 01:02 | 01:02 | 09:01 | 01:03 | 03:01 | 03:02 | 06:04 |
|  | 33:03 | 46:01 | 14:03 | 01:01 | 13:02 | 02:02 | 04:01 | 01:02 | 03:03 |

**Table S2. HLA-DP alleles of SW620, CFPAC-1, ASPC-1 cell lines**

| Cell lines | HLA-DP gene | Alleles |
| --- | --- | --- |
| SW620 | DPA1 | 01:03/02:01 |
|  | DPB1 | 01:01/04:01 |
| CFPAC-1 | DPA1 | 01:03/01:03 |
|  | DPB1 | 04:01/04:01 |

**Table S3. HLA restrictions of EBV-LCL cells**

| LCL lines | HLA-A | HLA-B | HLA-C | HLA-DRB1 | HLA-DQB1 | HLA-DPB1 |
| --- | --- | --- | --- | --- | --- | --- |
| D5 | 02:06/24:02 | 40:06/40:06 | 08:01/08:22 | 09:01/09:01 | ND | 02:01/02:01 |
| D12 | 24:02/24:02 | 40:01/48:01 | 07:02/08:03 | 04:05/15:01 | ND | 02:02/02:02 |
| B13 | 24:02/33:03 | 44:03/46:01 | 01:02/14:03 | 09:01/13:02 | 03:03/06:04 | 03:01/04:01 |
| B8 | 02:07/32:01 | 44:02/46:01 | 01:02/05:01 | 09:01/11:01 | 03:01/03:03 | 04:01/05:01 |
| B11 | 02:01/11:01 | 54:01/57:01 | 01:02/07:02 | 04:05/07:01 | ND | 04:02/05:01 |
| B4 | 02:07/33:03 | 46:01/58:01 | 01:02/03:02 | 03:01/09:01 | 02:01/03:03 | 05:01/05:01 |
| YY | 02:01/03:01 | 13:01/58:01 | 03:02/03:04 | 07:01/13:02 | ND | 02:02/09:01 |
| B10 | 02:07/11:01 | 38:02/46:01 | 01:03/07:02 | 08:02/12:02 | ND | 05:01/13:01 |
| B1 | 01:01/11:126 | 15:01/40:02 | 04:01/03:03 | 04:06/09:01 | 03:02/03:02 | 05:01/14:01 |
| D350 | 02:01/02:06 | 40:01/40:06 | 07:02/08:01 | 09:01/09:01 | ND | 02:01/17:01 |
| D2 | 02:01/02:07 | 46:01/46:01 | 01:02/01:02 | 09:01/09:01 | ND | 05:01/38:01 |
| WY | 11:01/11:01 | 15:02/55:02 | 07:02/08:01 | 12:02/14:54 | ND | 107:01/135:01 |

**Table S4. List of KRAS G12V-T15 epitopes replaced with alanine**

| N0. | Mutated amino acid | Amino acid sequences |
| --- | --- | --- |
| G12V-T15-A1 | T1A | AEYKLVVVGAVGV |
| G12V-T15-A2 | E2A | TAYKLVVVGAVGV |
| G12V-T15-A3 | Y3A | TEAKLVVVGAVGV |
| G12V-T15-A4 | K4A | TEYALVVVGAVGV |
| G12V-T15-A5 | L5A | TEYKAVVVGAVGV |
| G12V-T15-A6 | V6A | TEYKLAVVGAVGV |
| G12V-T15-A7 | V7A | TEYKLVAVGAVGV |
| G12V-T15-A8 | V8A | TEYKLVVAGAVGV |
| G12V-T15-A9 | G9A | TEYKLVVVAAVGV |
| G12V-T15-G10 | A10G | TEYKLVVVAGVGV |
| G12V-T15-A12 | G12A | TEYKLVVVGAVAV |
| G12V-T15-A13 | V13A | TEYKLVVVGAVGA |
